# Parents care needs with epileptic children: a hybrid model concept analysis

**DOI:** 10.1186/s12887-025-05595-8

**Published:** 2025-05-01

**Authors:** A. Khalili, F. Cheraghi, A. Fayyazi, A. R. Soltanian, Farshid Shamsaei

**Affiliations:** 1 Student Research Committee, School of Nursing and Midwifery, Hamadan University of Medical Sciences, Hamadan, Iran; 2 Chronic Diseases (Home Care) Research Center, Institute of Cancer, Avicenna Health Research Institute, Hamadan University of Medical Sciences, Hamadan, Iran; 3 Departments of Pediatric Nursing, School of Nursing and Midwifery, Hamadan University of Medical Sciences, Hamadan, Iran; 4 Autism Spectrum Disorders Research Center, Institute of Neuroscience and Mental Health, Avicenna Health Research Institute, Hamadan University of Medical Sciences, Hamadan, Iran; 5 Departments of Pediatrics, School of Medicine, Hamadan University of Medical Sciences, Hamadan, Iran; 6 Modeling of Noncommunicable Diseases Research Center, Institute of Health Sciences and Technologies, Avicenna Health Research Institute, Hamadan University of Medical Sciences, Hamadan, Iran; 7 Behavioral Disorders and Substance Abuse Research Center, Institute of Neuroscience and Mental Health, Avicenna Health Research Institute, Hamadan University of Medical Sciences, Hamadan, Iran; 8 Departments of Psychiatric Nursing, School of Nursing and Midwifery, Hamadan University of Medical Sciences, Hamadan, Iran

**Keywords:** Parents, Care needs, Epileptic child, Hybrid model

## Abstract

**Background:**

The care needs of epileptic children are a multidimensional concept that varies based on the experience and understanding of family caregivers. This study aimed to analyze the concept of parental care needs for children with epilepsy.

**Methods:**

This study was conducted using the hybrid method in three phases. In the theoretical phase, a systematic literature review was performed. In the fieldwork phase with a qualitative approach, 13 parents caring for epileptic children were investigated with individual and semi-structured interviews at the Besat Teaching-Treatment Center (Hamadan, Iran) in 2024. In the final phase, the concept of parental care needs was defined based on the findings of the theoretical and fieldwork phases.

**Results:**

The common antecedents identified in both the theoretical and fieldwork phases included low education levels and poor economic status. Additionally, the fieldwork phase highlighted the role of ineffective support systems. The common consequences were care quality improvement, financial burden reduction, care knowledge acquisition, and psychosocial status improvement, and the consequence of increasing stability in the family structure appeared in the fieldwork phase. The care needs of epileptic children’s parents included comprehensive support, therapeutic needs, and psychological needs, while financial constraints and inadequate care knowledge were identified as major challenges. These needs are influenced by factors such as low socioeconomic status and limited education, and their fulfillment leads to an improved quality of life and more effective disease management.

**Conclusion:**

Concentration on parental care needs and provision of appropriate support through education, financial resources, and social support can help reduce psychosocial pressures on families.

## Introduction

Epilepsy is a chronic neurological disorder in children, with a reported incidence of 4 to 8 cases per 1,000 children [1], and its prevalence is approximately 17.3 per 1,000 in developing countries [2]. In Iran, about 32.7% of epilepsy cases occur in individuals under 19 years of age [3]. Due to its chronic nature, epilepsy poses significant challenges to affected children in terms of physical, mental, and emotional health, as well as academic progress, which can greatly influence their quality of life and future prospects [1, 4, 5]. Additionally, 30–40% of individuals with epilepsy experience cognitive disorders, including difficulties with learning, memory, attention, and executive functioning [6].

The needs of children with epilepsy vary depending on factors such as disease severity, living environment, growth and developmental requirements, and cultural, physical, psychological, emotional, and educational factors. Consequently, parents of children with epilepsy face a wide range of emerging and multidimensional caregiving challenges, the fulfillment of which presents additional difficulties [7, 8, 9, 10].

However, caregiving challenges for children with epilepsy are further complicated by various factors, including parental resilience, coexisting conditions, socioeconomic status, family dynamics, and sociocultural influences [11]. In Iran, cultural and familial expectations play a significant role in caregiving. Extended family members often contribute to childcare, and societal norms emphasize parental responsibility in providing continuous support for children with chronic illnesses. However, stigma associated with epilepsy may limit access to adequate care and social support [12]. Given the diverse challenges faced by parents of children with epilepsy, their caregiving needs differ significantly [13]. Wo et al. reported that parents caring for children with epilepsy require information about the condition, continuous care, and access to parent support groups [5]. In a study by Jones et al., parents indicated that early-onset epilepsy and associated neurobehavioral problems significantly impacted family functioning, leading to increased restrictions on family activities and financial strain. Additionally, many parents reported difficulty accessing epilepsy specialists [10]. Similarly, Yu et al. highlighted the unmet care needs of caregivers, including excessive caregiving burdens, emotional distress, coping challenges, and a lack of adequate support [14].

In other words, the care needs of parents of children with epilepsy are multidimensional. Epilepsy not only affects the child but also has profound consequences for the entire family. Research indicates that parents of children with epilepsy experience heightened psychological distress, financial strain, and social isolation due to the unpredictability of seizures and the ongoing need for medical care. Additionally, families often struggle to balance caregiving responsibilities with work and social life, leading to increased emotional burden. Addressing these challenges requires a comprehensive understanding of parental care needs, which this study aims to explore using a hybrid model approach [15]. Family-centered care research has demonstrated that involving parents in the caregiving process enhances their sense of competence and improves their ability to fulfill their parental role. Given the emotional and practical burdens faced by parents of children with epilepsy, integrating family-centered care principles into epilepsy management may provide essential support, reduce caregiver stress, and ultimately improve the child’s well-being. Future studies should further examine the adaptability and effectiveness of such interventions in epilepsy care [16]. Therefore, gaining a meticulous and holistic understanding of these needs is the first step toward effectively addressing them. This understanding serves as a crucial foundation for identifying, planning, and fulfilling the care needs of parents of children with epilepsy [13, 17, 18].

Care encompasses the physical, emotional, and medical support provided to individuals with health conditions, ensuring their well-being and quality of life. Care needs refer to the various requirements and challenges caregivers face in fulfilling their responsibilities, including financial, psychological, informational, and social aspects [11].

Since no clear definition of parental care needs in epilepsy was identified in the existing literature, this study aims to refine and elaborate on this concept using a hybrid model. The hybrid concept analysis method was selected because it integrates theoretical insights from existing literature with empirical data from real-world experiences. Unlike purely theoretical or qualitative approaches, this method enables a more comprehensive and dynamic understanding of complex and evolving concepts, such as parental care needs. By combining a systematic literature review with fieldwork data, the hybrid approach ensures both conceptual clarity and practical relevance, making it a robust method for analyzing multifaceted healthcare-related concepts [19].

## Materials and methods

### Study design

Schwartz-Barcott and Kim (1986) proposed a hybrid model consisting of three sequential phases: (1) Theoretical Phase, (2) Fieldwork Phase, and (3) Final Analytical Phase. This model is widely used to refine and clarify less-explored concepts by integrating literature reviews with empirical findings. Each phase was structured as follows:

Theoretical Phase: A systematic literature review was conducted to identify existing definitions, attributes, antecedents, and consequences of parental care needs. The review followed the University of York guidelines and incorporated predefined inclusion and exclusion criteria, database searches, and quality appraisal. The extracted data were analyzed using conventional content analysis [20].

Fieldwork Phase: Based on the findings from the theoretical phase, semi-structured interviews were conducted with 13 parents of children with epilepsy at the Besat Teaching-Treatment Center (Hamadan, Iran). Thematic analysis was applied to identify patterns and categories emerging from participants’ experiences. Data collection continued until data saturation was achieved.

Final Analytical Phase: The findings from the theoretical and fieldwork phases were synthesized to refine and define the concept of parental care needs. Recurring themes from both phases were compared, leading to the development of an integrated definition [21].

### Theoretical phase

The inclusion criteria consisted of books and English/Farsi articles published in peer-reviewed journals that addressed the definitions, characteristics, facilitators, and inhibiting factors of parental care needs. Books related to parental care needs were also included in the review.

To ensure the quality of the selected studies, both qualitative and quantitative research were assessed using the Critical Appraisal Skills Program (CASP) for qualitative studies and the Strengthening the Reporting of Observational Studies in Epidemiology (STROBE) checklist for quantitative studies [19].

The literature search was conducted from January 2000 to December 2023 using the following Boolean search strings in various databases: (“epilepsy” OR “seizure disorder”) AND (“parental care” OR “care needs” OR “care burden”) AND (“children” OR “pediatric” OR “child health”) AND (“family support” OR “caregiver burden”) AND (“epilepsy management” OR “pediatric epilepsy”). Databases such as CINAHL, PubMed, Science Direct, OVID, Google Scholar, Magiran, and SID were systematically searched using these keywords along with their Persian equivalents. The searches were conducted independently by the first and second authors and verified by the third author. In addition to peer-reviewed journal articles, other sources included gray literature, conference proceedings, government reports, and unpublished research relevant to parental care needs in epilepsy. These sources were considered to ensure a comprehensive review of the available evidence.

Duplicate articles were excluded using EndNote software. After reviewing the abstracts, full-text articles that met the inclusion criteria were evaluated using appropriate assessment tools. The texts of the extracted articles and books were analyzed using the conventional content analysis approach based on the steps proposed by Graneheim & Lundman [22]. The text was carefully reviewed verbatim, line by line, and paragraph by paragraph multiple times until the authors achieved a general understanding. Relevant sections were extracted to identify meaning units and primary codes. The codes were read multiple times and categorized based on their similarities and relevance to the research concept. Independent codes were separated, and similar ones were merged into broader categories.

After second-level coding, the categories were compared, and those with similar characteristics were consolidated to form overarching categories. Efforts were made to ensure maximum intra-category homogeneity and inter-category heterogeneity. Finally, the categories were integrated into overarching themes. Data analysis was conducted using MAXQDA version 2018 [23]. The literature search continued throughout the fieldwork phase to supplement and validate findings.

**Fig. 1:**
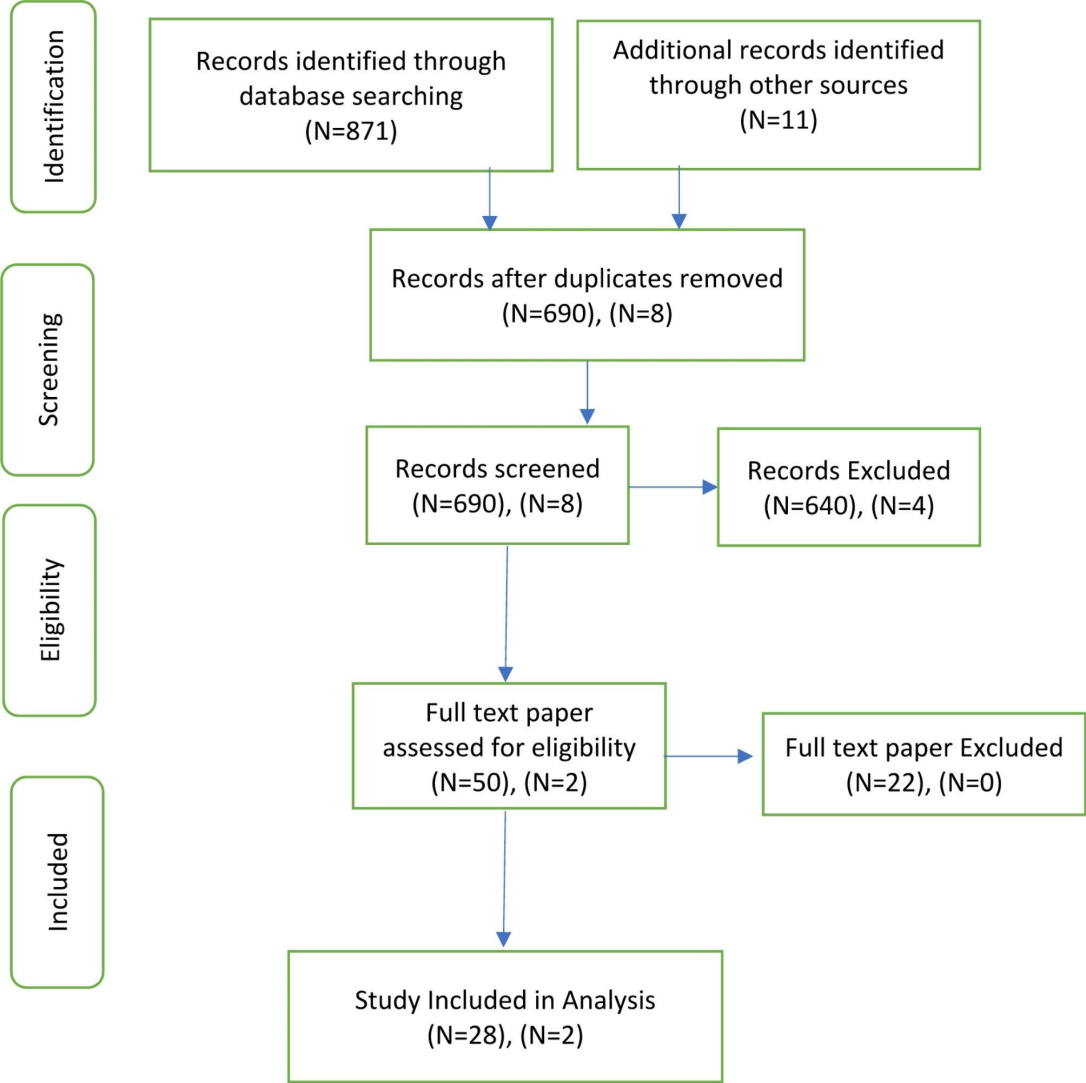
Study selection process

### Fieldwork phase

In this phase, 13 parents of children with epilepsy participated in face-to-face, semi-structured interviews conducted using a qualitative approach at Besat Hospital in Hamadan in 2024. Data collection was carried out over four months, from October 2024 to February 2025.

The interviews began with warm-up questions, such as: “Can you tell me a little about your child?” “How long has your child been diagnosed with epilepsy?” “What was your first reaction when you learned about your child’s condition?” “How would you describe a typical day in your life as a parent of a child with epilepsy?”

These were followed by exploratory questions, including: “What issues have you faced while caring for your child?” “What do you think can be done to meet your child’s care needs?” “What support do you expect to fulfill your child’s needs?” “How has caring for your child influenced your life?”

Participants were selected using purposive sampling and recruited until data saturation was reached. Inclusion criteria required participants to be parents of children diagnosed with epilepsy for at least six months, actively involved in their child’s care, and willing to participate in the study. Exclusion criteria included parents of children with severe comorbid conditions that significantly impacted their care needs (e.g., severe intellectual disability, neurodegenerative disorders) and those with limited involvement in their child’s daily caregiving.

Data saturation was determined when no new themes or codes emerged from the interviews, and further data collection no longer contributed to conceptual development. This was assessed through continuous comparison of interview transcripts and team discussions.

To ensure participant diversity, various factors were considered, including gender, age, education level, economic status, occupation, number of children, and ethnic background. Interviews were conducted in the head nurse’s office of the pediatric neurology ward, with each session lasting between 30 and 45 min, depending on participant interest. Interviews were digitally recorded, transcribed verbatim, and coded afterward.

Data analysis was conducted simultaneously with data collection using Conventional Content Analysis, following the approach proposed by Graneheim and Lundman (2004). Participants’ statements were examined to extract meaning units, which were read multiple times, coded, and categorized based on similarities and relevance to the research focus. These codes were then systematically reviewed, compared, and merged into broader categories. Next, the categories were analyzed, and those with similar characteristics were combined into overarching categories and subcategories. Throughout the process, codes and categories were continuously refined until the final stages of report preparation.

Qualitative data analysis was conducted using MAXQDA version 2018 [23]. The accuracy of the data was assessed using Lincoln and Guba’s criteria (credibility, dependability, transferability, and confirmability) [24].

### Final analytical phase

To validate and refine the definition of the intended concept in the analytical phase, the codes and categories derived from the fieldwork phase were compared with the data obtained from the literature review in the theoretical phase. Common characteristics of the concept of parental care needs were identified and reported in this phase.

## Results

In the theoretical phase, a total of 871 articles were identified through PubMed (127), OVID (115), Science Direct (386), CINAHL (94), and Google Scholar (149). After removing irrelevant and duplicate articles, 56 out of the remaining 118 abstracts were found to be highly relevant to care needs. Among these, only 30 studies specifically addressing the concept of “parental care needs” were selected for a detailed review to extract key elements necessary for defining and measuring comprehensive care (Fig. 1).

Table 1 provides a list of articles selected for analysis during the theoretical phase. These articles were reviewed to identify key aspects of holistic care and contributed to the development of the conceptual framework for this study.

**Table 1:** Articles selected for analysis during the theoretical phase

Row	Reference	Semantic Unit	Code
1	An Exploratory Study on Psychosocial Needs and Impacts of Pediatric Epilepsy on Caregivers. Kumar et al. 2021. India [17].	A child with epilepsy requires more care, time, and attention, all of which can lead to family dissatisfaction.	Caregiving burden due to epilepsy, which can impose costs on the family.
2	Anxiety among caregivers of children with epilepsy from western. Yang et al. 2020. China [25].	The young age of the child, poor financial status, and fear of recurrent seizures are issues that need attention.	Perceived support needs for caregivers while caring for a child with epilepsy.
3	Barriers to epilepsy care in Central Uganda, a qualitative interview and focus group study involving PLWE and their caregivers. Kaddumukasa et al. 2019. Uganda [26].	Addressing the lack of awareness, inadequate or incorrect knowledge, stigma, poor access, mental health conditions, and adherence to evidence-based treatments in patients with epilepsy is essential.	Care and management needs related to epilepsy, which are the primary source of parental stress.
4	Burden and Perceptions Associated with Epilepsy: Caregivers’ Perspectives. Nidhi Singh, 2015. India [27].	There is a need for a comprehensive healthcare system focused on mental health and support groups for caregivers to reduce stigma and anxiety associated with the disease.	Support needs to reduce the caregiving burden from the disease.
5	Burden of Seizure Clusters on Patients with Epilepsy and Caregivers, Penovich et al. 2017. USA [28].	Epilepsy education management and the availability of rescue treatments can potentially reduce the disease burden.	Need for education on managing epilepsy to reduce disease burden.
6	Caregivers of school children with epilepsy: findings of a phenomenological study. Roberts and Whiting, 2011. Canada [29].	Having a teacher experienced in working with children with epilepsy can help alleviate some of the challenges parents face.	Need for reassurance about the child’s well-being outside the home.
7	Caregiving in pediatric epilepsy: Results of focus groups and implications for research and practice. Smith et al. 2014. USA [30]	It’s understandable for parents to worry about continuously caring for a child with epilepsy to ensure they have a normal life.	Need for support to ensure a normal life for a child with epilepsy.
8	Concerns and Needs of Children with Epilepsy and Their Parents. McNELIS et al. 2007. USA [31].	Having initial information about the disease and obtaining therapeutic and social support to combat the challenges of caring for a child with epilepsy.	Need for therapeutic and social support to cope with challenges of caring for a child with epilepsy.
9	Effects of family-centered empowerment intervention on stress, anxiety, and depression among family caregivers of patients with epilepsy. Etemadifar, 2018. Iran [32].	Access to appropriate resources like awareness is crucial for improving care quality and enhancing self-management skills to boost caregiver efficacy for children with epilepsy.	Need for support and education to enhance care and self-efficacy for caregivers of children with epilepsy.
10	Epilepsy is associated with unmet health care needs compared to the general population despite higher health resource utilization-A Canadian population-based study. Reid, 2012. Canada [33].	Consulting with a health specialist, regular medical counseling, and unmet health needs are essential needs of parents of a child with epilepsy.	Unmet needs associated with caring for a child with epilepsy.
11	Evaluation of care burden and caregiving preparedness in caregivers of patients with epilepsy: A sample in eastern Turkey. Turan et al. 2021. Turkey [34].	The Turkish Language Institute defines care as “the act of caring, the effort to make something progress well or stay well, or the occupation of attending to one’s needs like feeding and dressing.”	Efforts to overcome challenges related to deviating from normality.
12	Exploring the needs and challenges of parents and their children in childhood epilepsy care: A qualitative study. Wo et al. 2018. Malaysia [5].	Parents’ main concerns include side effects of anti-epileptic drugs, epilepsy-related awareness, the child’s future, child’s self-esteem, or stigma.	Concerns arising for caregivers while caring for a child with epilepsy.
13	Unmet Supportive Care Needs Among Informal Caregivers of Patients with Cancer: Opportunities and Challenges in Informing the Development of Interventions. Lambert, 2017. Canada [35].	Caregiver needs fall into areas like comprehensive cancer care, emotional and psychological, impact on daily activities, relationships, information, and spirituality.	Supportive needs related to the responsibility of caring for a child with epilepsy.
14	Validation of the Needs Assessment of Family Caregivers-Cancer scale in an Asian population. Yang et al. 2020. USA [36].	Experiencing psychological, social, medical, and financial problems while caring for the patient.	Needs arising while caring for a child with epilepsy.
15	Measuring the Needs of Family Caregivers of People with Dementia. Bangerter et al. 2017. USA [37].	Unmet acute needs arise from recognizing a need and the subsequent realization that it is not satisfied by current services, support, or other resources.	Unmet needs beyond the caregivers’ capacity to care for a child with epilepsy.
16	Families’ experiences of living with pediatric epilepsy: A qualitative systematic review. Harden et al. 2016. UK [38].	Experiences like disappointment, worry, stress, personal growth, and gaining experience while caring for a child with epilepsy.	Needs for self-awareness and personal growth while caring for a child with epilepsy.
17	Knowledge, attitudes, and practices of caregivers of children with epilepsy in Sudan. Rahba et al. 2021. Sudan [7].	Essential awareness for caregivers to provide daily care to children with epilepsy.	Need for awareness to care for a child with epilepsy aiming for a normal life.
18	Lived experiences of caregivers of persons with epilepsy attending an epilepsy clinic at a tertiary hospital, eastern Uganda: A phenomenological approach. Okiah et al. 2022. Uganda [39].	Services and programs targeting epilepsy patients should consider the burden caregivers face in managing epilepsy.	Supportive needs related to caring for a child with epilepsy.
19	Parents’/caregivers’ fears and concerns about their child’s epilepsy: A scoping review. Carter et al. 2022. UK [40].	Emotional, psychological, and social concerns during care for a child with epilepsy.	Reducing emotional, psychological, and social concerns during care for a child with epilepsy.
20	Prevalence and Associated Factors of Mental Distress among Caregivers of Patients with Epilepsy in Ethiopia. Seid et al. 2018. Ethiopia [41].	Social, emotional, behavioral, and financial challenges while caring for a child with epilepsy.	Supportive needs related to caring for a child with epilepsy.
21	Psychosocial care needs of the parents having children with epilepsy. Rajalakshmi, 2014. India [42].	Need for necessary awareness to manage children’s epilepsy.	Need for necessary awareness to manage epilepsy.
22	Seizure detection at home: Do devices on the market match the needs of people living with epilepsy and their caregivers? Bruno et al. 2020. UK [43].	Future efforts should focus on demonstrating the validity and utility of collected data in the daily lives of caregivers of people with epilepsy to meet their needs and ensure quality of life improvement.	Needs for safety improvement, better clinical management, and assurance about the child’s future condition with epilepsy.
23	Continuing Psychosocial Care Needs in Children with New-Onset Epilepsy and Their Parents. Shore et al. 2009. USA [44].	Existence of psychosocial care needs that can have a more significant negative impact on family life.	Supportive needs related to caring for a child with epilepsy.
24	Stressors of Caregivers of School-Age Children with Epilepsy and Use of Community Resources, Gladys Saburi, 2011. Zimbabwe [45].	Need for support and awareness of information that could reduce parental stress and better equip them to meet the emotional needs of a child with epilepsy.	Need for support to meet the emotional needs of a child with epilepsy.
25	The experiences of caregivers of children with epilepsy: A meta-synthesis of qualitative research studies. Yu et al. 2022. China [14].	Unmet care demands leading to very serious emotional distress for parents.	Unmet needs related to caring for a child with epilepsy, causing emotional distress to parents.
26	The information needs of careers of adults diagnosed with epilepsy. Kendall et al. 2003. UK [46].	The necessity for caregivers to have the required awareness and information in an understandable way to care for a child with epilepsy.	Unmet needs by parents while caring for a child with epilepsy.
27	The information needs of parents of children with early-onset epilepsy: A systematic review. Nevin, 2020. Australia [4].	Specific need for awareness regarding comorbidities with epilepsy and how to provide emotional support for the child with epilepsy by parents.	Informational needs for parents to manage the child’s epilepsy appropriately.
28	The Needs and Problems in Epilepsy Caregiving: A Qualitative Exploration. Lua et al. 2015. Malaysia [47].	Need for caregiving, psychological, and financial support when caring for a child with epilepsy.	Supportive needs related to caring for a child with epilepsy.
29	Intense parenting: a qualitative study detailing the experiences of parenting children with complex care needs. Woodgate, 2015. Canada [48].	There is a need for more support and resources for caring for children with complex care needs.	Unmet needs related to caring for a child with epilepsy.
30	Care for the Caregivers: A Review of Self-Report Instruments Developed to Measure the Burden, Needs, and Quality of Life of Informal Caregivers. Deeken et al. 2003. USA [49].	Needs are “problems related to health status and problems related to the quality of health care, provided that both can generate the need for more professional care.”	Perceived needs that motivate caregivers while caring for a child with epilepsy to improve health status and care quality.

Based on the literature review, the characteristics of parental care needs were categorized into six key dimensions: the need for comprehensive support, financial challenges of care, limited care-related knowledge, emerging treatment needs, efforts to facilitate the caregiving process, and psychological challenges of caregiving. These dimensions highlight the various aspects of parental care needs, encompassing both practical and emotional challenges faced by caregivers.

Table 2 provides examples of meaning units representing these characteristics, extracted from the literature along with their corresponding codes.

**Table 2:** Examples of meaning units representing the characteristics of parental care needs extracted from the literature along with their codes

Codes	The meaning unit
The need for comprehensive support	Medical personnel should pay more attention to caregivers with younger ages, poor financial status, and parents with more fear of epilepsy.
Financial challenges of care	Caring for an epileptic child incurs a financial drain on families due to the costs of medication, medical care, and other miscellaneous special needs.
Low care knowledge	Epileptic children’s parents reported a high need for information, support, and reducing concerns about unlikely events such as brain damage and death.
Emerging therapeutic needs	Epilepsy development increases the need for specialist consultation, regular medical consultation, hospitalization, and unmet health needs.
Efforts to facilitate the care process	The caregiving burden due to epilepsy can appear as job loss or inability to work, loss of household income, family burden for caregiving with further reduced wages, and medication costs.
Psychological challenges of care	Caregivers of epileptic children experience severe emotional, physical, and economic burdens due to the nature, chronicity, inability, and epilepsy-associated stigma.

The antecedents and consequences of parental care needs were identified from the reviewed literature. The antecedents included low education levels and poor economic status, which were found to be key factors influencing parental caregiving challenges. The consequences of addressing these needs included improved care delivery, reduced financial burden, enhanced care-related knowledge, more effective disease management, better quality of life, and improved psychosocial well-being.

### Antecedents

Low education level: A proper understanding of the disease by family members is essential for effective patient care and condition management. Without adequate knowledge, caregivers may struggle with decision-making, leading to adverse outcomes. Therefore, access to appropriate resources is crucial to improving care quality and self-management skills, ultimately enhancing the self-efficacy of caregivers of children with epilepsy [32].

Poor economic status: Parents identified financial constraints as a primary source of stress, particularly in meeting their child’s basic needs and covering essential medical expenses [26]. Therefore, caring for a child with epilepsy places a significant financial burden on the family due to medication costs, medical care expenses, and other specialized needs [17].

### Consequences

Proper recognition of parents’ care needs can enhance care provision, knowledge acquisition, effective disease management, quality of life, and psychosocial well-being, while also reducing the financial burden of care [18, 50, 51].

Definition of parental care needs in the theoretical phase.

Based on the literature review and care needs analysis, parental care needs can be defined as a multidimensional concept arising from the challenges of pediatric epilepsy and the demands of caregiving. This concept encompasses several key dimensions, including the need for comprehensive support, financial challenges, limited care-related knowledge, emerging treatment needs, efforts to facilitate the caregiving process, and psychological challenges.

Socioeconomic factors, such as poor economic status and low education levels, significantly influence parental care needs. Recognizing and addressing these needs can lead to improved care provision, enhanced knowledge acquisition, effective disease management, better quality of life, and improved psychosocial well-being, while also reducing the financial burden of care.

### Fieldwork phase

The study included 13 parents (4 fathers and 9 mothers) of children with epilepsy (7 boys and 6 girls). The mean age of the parents was 42.6 ± 5.38 years, while the mean age of the children was 9.75 ± 2.24 years. The average duration of epilepsy among the children was 5 years. Additional demographic characteristics of the participants are presented in Table 3.

**Table 3:** Participants’ demographic characteristics in the fieldwork phase

Row	Parent Gender	Parent Age	Child Age (Years)	Child Gender	Duration of Illness (Years)	City of Residence	Parent Occupation	Parent Education Level
1	Female	42	9.5	Male	7.5	Hamadan	Homemaker	Primary School
2	Female	39	6.5	Male	5.5	Hamadan	Homemaker	Illiterate
3	Male	46	11	Male	4	Hamadan	Homemaker	Primary School
4	Female	45	12	Female	8	Hamadan	Homemaker	Primary School
5	Female	34	5.5	Female	5	Hamadan	Homemaker	High School Diploma
6	Male	48	12	Male	1.5	Kurdistan	Self-Employed	Bachelor’s Degree
7	Female	38	10	Male	1.5	Hamadan	Homemaker	High School Diploma
8	Male	44	12	Male	4	Kurdistan	Self-Employed	Below High School
9	Female	42	11	Female	5	Hamadan	Homemaker	Below High School
10	Female	46	8	Female	5	Hamadan	Homemaker	Below High School
11	Female	44	11	Female	8	Kurdistan	Homemaker	Illiterate
12	Male	44	10	Male	2	Hamadan	Self-Employed	Below High School
13	Female	39	7	Female	5	Hamadan	Homemaker	High School Diploma

**Table 4:** Categories and subcategories extracted from the interviews conducted in the fieldwork phase

Categories	Subcategories	Examples of codes
Insufficient knowledge about the disease	Poor disease management	I don’t know much about epilepsy, nor any information about it, nobody told us anything (Participant 12).
	Inadequate knowledge of care delivery	
	Low information on the disease	
	Low information on the nature of the disease	
Increased financial burden due to epilepsy	Limited care ability because of financial problems	We have always had a problem with the costs of drugs. At first, my husband did not buy the drugs at all and always fought with the doctor. When we visited the doctor’s office, the visit cost was very high, the brain scan cost was very high, and we always had problems; they did not give a discount (Participant 1).
	The need for financial support	
	Struggle to afford costs	
	Limited financial ability	
	High treatment costs	
The need for support	The need for support from other organizations	I would like to have a place for mothers and children who have epilepsy, where people would go to them. When I see my own gender with the same suffering and the same problem I seem to feel more relaxed. (Participant 5).
	Difficult access to drugs	
	Continuous need for treatment follow-up	
	The need for understanding from the spouse	
	The need for advice	
Caregivers’ psychological concerns	Desperate to improve conditions	We always worry that anything might happen God forbid because a similar case was in our village who died, I am too worried that the same thing might happen to my child. I always have a guilty conscience. I wish I could have taken more care of my child before not to see like this (Participant 2).
	Constant guiltiness	
	The inner torment of having a sick child	
	Occurrence of stress reactions in the mother	
	Mental exhaustion of parents	
	Tension in siblings	
Vulnerable family	The need for high patience	I begged God for high patience, I say, God, give me patience to bear this way, not to beat, not to bother, and not to stretch out my hand to bat this child (Participant 11).
	Unwanted changes in the family atmosphere	
	The need for a calm atmosphere in the family	
	Trying to maintain stability in the family	
Concerns related to the child’s physical health	Fear of unwanted happenings to the child	Wherever my child goes, he/she wants me to follow him/her. For example, when he/she goes to school, I take him/her, and I follow him/her wherever he/she goes.
	Fear of the unknown	
	Concerns about the child’s condition	

### Antecedents and consequences of the fieldwork phase

The literature review findings indicated that low education levels and unstable economic status were key contributors to parental care needs. The fieldwork phase further revealed that ineffective support systems also serve as antecedents that can impact parental care needs. Specifically, families with limited financial resources and insufficient external support face greater challenges in meeting caregiving demands.

A 44-year-old mother described her experience, stating:

*“At that time*,* goods were affordable—my father could buy things for him/her*,* and my spouse could also afford it. But now*,* my spouse can no longer afford to provide for her/him. The welfare system says they pay her a salary*,* but it’s only 600 tomans per month.”*

### Consequences of the fieldwork phase

Proper recognition of parental care needs can lead to essential knowledge acquisition about the disease, reduction of epilepsy-related financial burden, access to necessary medical support, less reliance on relatives and friends for assistance, decreased psychological distress among caregivers, greater independence in performing personal activities, and enhanced family stability.

In addition to the findings from the literature review, the fieldwork phase identified increased family stability as a key consequence of addressing parental care needs. Greater patience among family members, a more supportive family atmosphere, and a sense of calm within the household were all reported as contributing factors to family stability.

A 45-year-old mother shared her experience, stating:

*“Our family gatherings feel disconnected—everyone is tired*,* no one laughs*,* there’s no entertainment*,* no relatives*,* nothing. Everyone keeps to themselves. My son comes home*,* eats*,* and then goes straight to his room*,* while we sit in the living room*,* feeling bored.”*

### Definition of the concept of caregiving for sick children

Caregiving for children with chronic illnesses, including epilepsy, is a multifaceted process that involves providing physical, emotional, and medical support. It requires managing treatment adherence, addressing financial and psychological burdens, and ensuring a stable and nurturing environment for the child. Parents play a central role in coordinating healthcare services and coping with the long-term challenges of caregiving.

In the case of epilepsy, parental caregiving responsibilities extend beyond routine medical care, as they must navigate complex emotional, financial, and social challenges. The specific care needs of parents of children with epilepsy can be categorized as follows.

### Definition of parental care needs in the fieldwork phase

Based on the fieldwork findings and care needs analysis, parental care needs can be defined as a multidimensional concept that affects all aspects of family life. It encompasses comprehensive support, anxiety and emotional turmoil, and the role of the family in caring for the child (Table 4).

Several factors influence this concept, including low education levels, unstable economic conditions, and a lack of adequate support systems to meet parents’ care needs. Recognizing and addressing these needs can lead to essential knowledge acquisition about epilepsy, reduced financial burden, access to necessary medical support, decreased reliance on external assistance, reduced psychological distress among caregivers, greater independence in daily activities, and enhanced family stability.

### Final analysis phase

In this phase, the antecedents and consequences identified in the theoretical phase were compared with those from the fieldwork phase. Based on these findings, the concept of parental care needs was refined and defined.

### Comparison of the antecedents of the concept of parental care needs between the theoretical and fieldwork phases

The antecedents identified in the theoretical phase included low education levels and poor economic status, while those in the fieldwork phase included low education levels, unstable economic status, and ineffective support systems. Therefore, low education levels and economic challenges were common findings in both phases. However, the issue of ineffective support systems was not mentioned in the literature review and emerged specifically in the fieldwork phase.

This finding suggests that parents of children with epilepsy require support from their companions, yet they often do not receive adequate assistance from them.

### Comparing the consequences of the concept of parental care needs between the theoretical and fieldwork phases

The results of the theoretical phase included improvements in care delivery, a reduction in the financial burden of care, enhanced acquisition of care-related knowledge, more effective disease management, improved quality of life, and better psychosocial well-being. The fieldwork phase yielded similar outcomes, including acquiring essential knowledge about the disease, reducing the financial burden associated with epilepsy, securing necessary medical support, decreasing reliance on companions for assistance, alleviating caregivers’ psychological concerns, fostering greater independence in performing personal activities, and enhancing family stability.

All the outcomes of the theoretical phase completely overlapped with those of the fieldwork phase. However, in addition to these shared consequences, an increase in family stability emerged specifically in the fieldwork phase. This finding suggests that addressing the care needs of parents with epileptic children fosters a more harmonious family environment, strengthens emotional connections among family members, and ultimately contributes to maintaining family stability.

In the final analytical phase, these findings were further synthesized, confirming that addressing parental care needs results in multiple positive outcomes, including improved care quality, a reduced financial burden, increased parental knowledge, enhanced disease management, and greater family stability. This phase reinforced the significance of emotional and psychological well-being in caregivers, emphasizing the need for holistic support systems for families of children with epilepsy.

### Analysis and comparison of the definition of parental care needs between the theoretical and fieldwork phases

A comparison of these two definitions reveals that the theoretical phase definition encompasses dimensions such as emerging therapeutic needs and psychological challenges. However, the fieldwork phase definition introduces an additional dimension, including anxiety and emotional turmoil, which has more practical implications for the daily lives of families, alongside the dimensions identified in the theoretical phase. Consequently, the definitions of parental care needs from the theoretical and fieldwork phases were integrated to formulate the final analytical definition of parental care needs for a child with epilepsy, as described below.

The care needs of parents of children with epilepsy are a multidimensional psychological phenomenon that impacts all aspects of family life following the child’s diagnosis and the subsequent need for care. These needs encompass various dimensions, including comprehensive support, financial challenges, limited care-related knowledge, emerging medical needs, efforts to facilitate the care process, and psychological challenges. Several factors influence these needs, such as poor economic status, low education levels, and a lack of capable supporters. Addressing and fulfilling these needs can enhance care quality, reduce financial burdens, improve care-related knowledge, facilitate more effective disease management, enhance the quality of life for both the child and parents, foster greater independence in personal activities, alleviate psychological concerns, and promote stability within the family structure.

## Discussion

Our research highlights that the care needs of parents of children with epilepsy are complex and multifaceted, encompassing elements such as comprehensive support, financial challenges, knowledge deficits, emerging medical requirements, efforts to ease caregiving tasks, and psychological burdens. Socioeconomic status, educational background, and the availability of support systems play crucial roles in shaping these needs. Addressing these factors can improve care quality, alleviate financial pressures, optimize disease management, and stabilize family life.

Our findings align with previous research. Okiah et al. categorized caregivers’ needs into psychological, socioeconomic, and physical domains [52]. Yu et al. identified that caregivers face significant burdens, emotional challenges, and a need for coping mechanisms [14], while Zhang et al. examined the prevalence of anxiety, depression, sleep disturbances, and family dysfunction among caregivers [53], all underscoring the necessity of a holistic approach.

One crucial finding is the importance of accessible information in supporting caregivers’ psychological well-being. Parents expressed a strong demand for clear, practical, and condition-specific information about epilepsy, particularly regarding associated health issues and emotional support. The absence of such information often leads to heightened uncertainty, stress, and social isolation as caregivers struggle to manage their child’s condition. Nevin et al. observed similar patterns, noting that limited access to information exacerbates caregivers’ emotional strain, increasing anxiety and exhaustion [4].

The psychological impact of caregiving is closely linked to informational support. Participants in our study frequently reported feelings of guilt, anxiety, and social withdrawal, largely due to uncertainties about their child’s condition and a lack of professional guidance. Studies by Wo et al. and Hussain et al. reinforce these findings, emphasizing the necessity of ongoing education, continuous care, and support networks to alleviate psychological distress [5, 54]. McNelis et al. have also highlighted caregivers’ struggles and the urgent need for reliable information [31].

Additionally, our findings advocate for family-centered support services as an essential resource for easing the caregiving burden. Echoing Jones et al., who emphasized the importance of tailored pediatric epilepsy services [10], our study underscores the need for services that consider neurobehavioral aspects and promote family cohesion. O’Toole et al. further identified family relationship strain as a significant issue, pointing to the necessity of emotional support within the home [55]. Moreover, the social challenges surrounding epilepsy disclosure, as discussed by Kampra et al., reflect the persistent stigma and lack of social guidance [56].

By integrating these dimensions into a coherent framework, our study provides a deeper understanding of parental care needs, advocating for interventions that collectively address psychological, financial, and informational challenges.

Our findings call on policymakers and healthcare providers to develop targeted support systems for parents of children with epilepsy, focusing on financial relief, educational resources, psychological support, and community engagement to enhance caregiver well-being and strengthen family dynamics.

### Limitations of the study

This study has several limitations. The hybrid model used may lead to varying and subjective interpretations, as it relies on qualitative analysis. Additionally, cultural and social factors influencing parental care needs were not explored, as they were beyond the researchers’ control. Furthermore, the study focused solely on parents’ perspectives and does not provide a comprehensive representation of other stakeholders’ views. Future research should incorporate healthcare providers’ perspectives to gain a more holistic understanding of the issue.

## Conclusion

Through its hybrid approach, this study combines literature review with fieldwork data to offer new insights into the care needs of parents of children with epilepsy. Accurately recognizing these needs can yield several positive outcomes, including improved caregiver knowledge and access to information, reduced financial strain, enhanced disease management, better care quality, and strengthened mental health among caregivers. Addressing these needs not only improves the quality of life for children with epilepsy but also reinforces family stability.

By prioritizing parental care needs and offering support through educational initiatives, financial aid, and social networks, we can significantly reduce psychosocial pressures on families, thereby improving both the quality of care and overall life satisfaction for all family members involved.

## Data Availability

All data generated or analyzed during this study are included in this published article. Additional information is available from the corresponding author upon reasonable request.
